# Longitudinal Assessment of Resident Performance Using Entrustable Professional Activities

**DOI:** 10.1001/jamanetworkopen.2019.19316

**Published:** 2020-01-15

**Authors:** Daniel J. Schumacher, Daniel C. West, Alan Schwartz, Su-Ting Li, Leah Millstein, Elena C. Griego, Teri Turner, Bruce E. Herman, Robert Englander, Joni Hemond, Valera Hudson, Lauren Newhall, Kenya McNeal Trice, Julie Baughn, Erin Giudice, Hannah Famiglietti, Jonathan Tolentino, Kimberly Gifford, Carol Carraccio

**Affiliations:** 1 Department of Pediatrics, Cincinnati Children’s Hospital Medical Center, Cincinnati, Ohio; 2 University of Cincinnati College of Medicine, Cincinnati, Ohio; 3 Department of Pediatrics, Children’s Hospital of Philadelphia, Philadelphia, Pennsylvania; 4 Department of Pediatrics, University of Pennsylvania, Philadelphia; 5 Department of Medical Education, University of Illinois at Chicago; 6 Association of Pediatric Program Directors Longitudinal Educational Assessment Research Network, McLean, Virginia; 7 Department of Pediatrics, University of Illinois at Chicago; 8 Department of Pediatrics at the University of California Davis Health, Sacramento; 9 Department of Pediatrics, University of Maryland School of Medicine, Baltimore; 10 Department of Pediatrics, Seattle Children’s Hospital, Seattle, Washington; 11 Department of Pediatrics, Texas Children’s Hospital/Baylor College of Medicine, Houston; 12 Department of Pediatrics, University of Utah, Salt Lake City; 13 Department of Pediatrics, University of Minnesota Medical School, Minneapolis; 14 Department of Pediatrics, Children’s Hospital of Georgia/Augusta University, Augusta; 15 Department of Pediatrics, University of North Carolina, Chapel Hill; 16 Department of Pediatrics, Mayo Medical School, Rochester, Minnesota; 17 Department of Pediatrics, New York University, New York; 18 Department of Pediatrics, Stony Brook University, Stony Brook, New York; 19 Department of Internal Medicine, Stony Brook University, Stony Brook, New York; 20 Department of Pediatrics, Dartmouth University, Lebanon, New Hampshire; 21 American Board of Pediatrics, Chapel Hill, North Carolina

## Abstract

**Importance:**

Entrustable professional activities (EPAs) are an emerging workplace-based, patient-oriented assessment approach with limited empirical evidence.

**Objective:**

To measure the development of pediatric trainees’ clinical skills over time using EPA-based assessment data.

**Design, Setting, and Participants:**

Prospective cohort study of categorical pediatric residents over 3 academic years (2015-2016, 2016-2017, and 2017-2018) assessed on 17 American Board of Pediatrics EPAs. Residents in training at 23 pediatric residency programs in the Association of Pediatric Program Directors Longitudinal Educational Assessment Research Network were included. Assessment was conducted by clinical competency committee members, who made summative assessment decisions regarding levels of supervision required for each resident and each EPA. Data were collected from May 2016 to November 2018 and analyzed from November to December 2018.

**Interventions:**

Longitudinal, prospective assessment using EPAs.

**Main Outcomes and Measures:**

Trajectories of supervision levels by EPA during residency training and how often graduating residents were deemed ready for unsupervised practice in each EPA.

**Results:**

Across the 5 data collection cycles, 1987 residents from all 3 postgraduate years in 23 residency programs were assigned 25 503 supervision level reports for the 17 general pediatrics EPAs. The 4 EPAs that required the most supervision across training were EPA 14 (quality improvement) on the 5-level scale (estimated mean level at graduation, 3.7; 95% CI, 3.6-3.7) and EPAs 8 (transition to adult care; mean, 7.0; 95% CI, 7.0-7.1), 9 (behavioral and mental health; mean, 6.6; 95% CI, 6.5-6.6), and 10 (resuscitate and stabilize; mean, 6.9; 95% CI, 6.8-7.0) on the expanded 5-level scale. At the time of graduation (36 months), the percentage of trainees who were rated at a supervision level corresponding to “unsupervised practice” varied by EPA from 53% to 98%. If performance standards were set to align with 90% of trainees achieving the level of unsupervised practice, this standard would be met for only 8 of the 17 EPAs (although 89% met this standard for EPA 17, performing the common procedures of the general pediatrician).

**Conclusions and Relevance:**

This study presents initial evidence for empirically derived practice readiness and sets the stage for identifying curricular gaps that contribute to discrepancy between observed practice readiness and standards needed to produce physicians able to meet the health needs of the patient populations they serve. Future work should compare these findings with postgraduation outcomes data as a means of seeking validity evidence.

## Key Points

**Question:**

What is the progression of performance for entrustable professional activities (EPAs) throughout pediatric residency training and at graduation?

**Findings:**

This multisite cohort study of 1987 pediatric residents found that developmental growth curves can be established for EPAs. When generated to reflect the results in this study, at least 90% of trainees achieved the level of unsupervised practice at the end of residency for only 8 of the 17 EPAs studied.

**Meaning:**

This study suggests that gaps exist between observed practice readiness and standards needed to produce physicians able to meet the health needs of the patient populations they serve based on the general pediatrics EPAs.

## Teaser

This cohort study measures the development of pediatric resident clinical skills using assessments based on entrustable professional activities.

## Introduction

Medical education throughout much of the world has been transitioning to a competency-based medical education (CBME) system.^1,2,3,4,5^ The core tenet of CBME is that individuals advance through training at variable rates when they demonstrate sufficient skill in a defined set of competencies, rather than at predetermined points during the training process.^6,7,8^ In this framework, competencies provide a common understanding of the desired outcomes of training for learners and teachers, while informing curricula and assessment across the continuum of medical education.^4,9,10,11^ Over the past 2 decades, competency models have been developed and implemented to variable extents throughout the world.^1,2,3,12^ In the United States, the primary graduate medical education (GME) model is the milestones developed by the Accreditation Council for Graduate Medical Education and the American Board of Medical Specialties.^1^ Since 2014, the Accreditation Council for Graduate Medical Education has required GME training programs to periodically measure and report ratings of specialty-specific competency milestones for individual trainees, but does not yet require the milestones be used to make advancement decisions.^1,13,14^

Entrustable professional activities (EPAs) were introduced to better inform advancement decisions by focusing on the essential professional activities a physician practicing in a particular specialty or subspecialty should be able to perform (eg, care for a well newborn in pediatrics or perform a cesarean delivery in obstetrics).^11,15,16^ They synthesize the numerous, more abstract, context-independent competencies and milestones into a smaller number of observable activities, providing clinical context that is necessary for meaningful assessment. Entrustment decisions, which are intended to be workplace-based assessments made at the frontline of care, are based on the perceived amount of supervision a trainee requires to safely and effectively perform a professional activity. With EPAs, assessors take advantage of a wide-angle lens to view learners, integrating the competencies required to perform the EPA. Finally, EPAs focus on the ability to provide safe and high-quality care, bringing the patient into the assessment equation.

Many EPA frameworks have been developed for different medical specialties and levels of trainees worldwide.^11,17,18,19,20,21,22,23,24^ There is limited but promising evidence that EPAs can be used to assess trainees and make advancement decisions on a small scale in both undergraduate medical education and GME.^25,26,27,28,29,30^ However, it is unknown whether EPAs can be implemented on a large scale in diverse clinical training environments to measure the progress of trainees over time or be used to make summative, end-of-training decisions about readiness to practice outside of a training environment.^25,31,32,33^ Filling this gap is important given the potential of EPAs to serve as a CBME framework that is intuitive to trainees and assessors and could guide curricula and assessment across the continuum of medical education to produce physicians who better meet the needs of society.^9,34,35^

Seeking to take a significant step toward filling the described gaps, we conducted a multisite study at diverse pediatric residency programs to (1) gather preliminary data on EPA-based assessment on a large scale, hypothesizing that the development of trainees’ clinical skills would increase over time but vary by EPA; (2) test the validity argument of the EPA assessment model, hypothesizing that the level of trainee supervision required for each EPA for each resident decreases over time; and (3) determine baseline data on the mean level of performance (supervision level required) that residents demonstrate by the end of training on each of the 17 general pediatrics EPAs to begin to standardize entrustments to make end-of-training decisions on readiness to practice.

## Methods

### Study Design

Over the 2015 to 2016, 2016 to 2017, and 2017 to 2018 academic years, clinical competency committees (CCCs) at participating programs reported supervision levels assigned to their residents for 5 to 6 of the American Board of Pediatrics general pediatrics EPAs^11^ (Table 1) twice yearly (fall and spring), with the exception of the first academic year included in the study (which included spring-only data).^36^

**Table 1.** EPAs and Related Supervision Levels

EPA	Supervision Levels
EPA 1: Provide consultation to other health care providers caring for children	Level 1: Trusted to observe only Level 2: Trusted to execute with direct supervision and coaching Level 3: Trusted to execute with indirect supervision and discussion of information conveyed for most simple and some complex cases Level 4: Trusted to execute with indirect supervision and may require discussion of information conveyed but only for selected complex cases Level 5: Trusted to execute without supervision
EPA 2: Provide recommended pediatric health screening EPA 3: Care for the well newborn EPA 4: Manage patients with acute, common diagnoses in an ambulatory, emergency, or inpatient setting EPA 5: Provide a medical home for well children of all ages (entrustment decisions for this EPA may require stratification by age group) EPA 6: Provide a medical home for patients with complex, chronic, or special health care needs (entrustment decisions for this EPA may require stratification by age group) EPA 7: Recognize, provide initial management and refer patients presenting with surgical problems EPA 8: Facilitate the transition from pediatric to adult health care EPA 9: Assess and manage patients with common behavior/mental health problems EPA 10: Resuscitate, initiate stabilization of the patient and then triage to align care with severity of illness (entrustment decisions for this EPA may require stratification by two age groups: neonate and non-neonate) EPA 17: Demonstrate competence in performing the common procedures of the general pediatrician	Level 1: Trusted to observe the EPA Level 2: Trusted to practice EPA only under proactive, full supervision as a coactivity with the supervisor Level 3: Trusted to practice EPA only under proactive, full supervision with the supervisor in the room and ready to step in as needed Level 4: Trusted to practice EPA only under reactive, on-demand supervision with supervisor immediately available and ALL findings double checked Level 5: Trusted to practice EPA only under reactive, on-demand supervision with supervisor immediately available and KEY findings double checked Level 6: Trusted to practice EPA only under reactive, on-demand supervision with supervisor distantly available (eg, by phone), findings reviewed Level 7: Trusted to practice EPA unsupervised Level 8: Trusted to supervise others in practice of EPA (where supervision means: ability to assess patient and learner needs ensuring safe, effective care and further trainee development by tailoring supervision level)
EPA 11: Manage information from a variety of sources for both learning and application to patient care	Level 1: Trusted to perform with direct supervision Level 2: Trusted to perform with indirect supervision with supervisor checking findings Level 3: Trusted to perform with indirect supervision with supervisor available for requested help Level 4: Trusted to perform without supervision Level 5: Trusted to supervise others
EPA 12: Refer patients who require consultation	Level 1: Trusted to observe only Level 2: Trusted to execute with direct supervision and coaching Level 3: Trusted to execute with indirect supervision and discussion of information conveyed for most simple and some complex cases Level 4: Trusted to execute with indirect supervision and may require discussion of information conveyed but only for selected complex cases Level 5: Trusted to execute without supervision
EPA 13: Contribute to the fiscally sound, equitable and collaborative management of a health care workplace	Level 1: Trusted to observe only Level 2: Trusted to perform with direct supervision and coaching with supervisor verifying work product for accuracy Level 3: Trusted to perform with supervisor serving as a consultant for all tasks Level 4: Trusted to perform with supervisor serving as a consultant but only for complex tasks Level 5: Trusted to perform without supervision
EPA 14: Apply public health principles and quality improvement methods to improve population health	Level 1: Trusted to observe only Level 2: Trusted to contribute with direct supervision and coaching as a member of a collaborative effort to improve care at the institutional level Level 3: Trusted to contribute without direct coaching as a member of a collaborative effort to improve care at the institutional level Level 4: Trusted to lead collaborative efforts to improve care for populations and systems at the institutional level Level 5: Trusted to lead collaborative efforts to improve care at the level of populations and systems at the regional and/or national level
EPA 15: Lead an interprofessional health care team	Level 1: Trusted to participate only Level 2: Trusted to lead with direct supervision and coaching Level 3: Trusted to lead with supervisor occasionally present to provide advice Level 4: Trusted to lead without supervisor present but requires coaching to improve member and team performance Level 5: Trusted to lead without supervision to improve member and team performance
EPA 16: Facilitate handovers to another health care provider either within or across settings	Level 1: Trusted to observe only Level 2: Trusted to execute with direct supervision and coaching Level 3: Trusted to execute with indirect supervision with verification of information after the handover for most simple and some complex cases Level 4: Trusted to execute with indirect supervision and verification of information after the handover for selected complex cases Level 5: Trusted to execute without supervision

Abbreviation: EPA, entrustable professional activity.

The institutional review board (IRB) at Cincinnati Children’s Hospital Medical Center (lead site) granted exempt status to this study. The IRB at each participating institution also reviewed and approved or exempted this study. A waiver of documented consent was granted at the lead site as this research presented no more than minimal risk of harm to participants and involved no procedures for which written consent is normally required outside of the research context. The individual IRB at each site made its own determination regarding documented consent or waiver of documented informed consent. This study followed the Strengthening the Reporting of Observational Studies in Epidemiology (STROBE) reporting guideline.

### Setting and Participants

We recruited CCCs at 23 pediatrics residency programs (eTable 1 in the Supplement) from the Association of Pediatric Program Directors’ Longitudinal Educational Assessment Research Network based on range of size and geographic distribution.^37^ All 132 network sites at the time of study inception were invited to participate. All 3 years of categorical (ie, not combined training program, such as medicine/pediatrics) pediatric residents at participating sites were eligible for inclusion in the study.

### Variables

All 17 general pediatrics EPAs were assessed using supervision scales (Table 1). We used 2 separate scales in an effort to bridge the education-training-practice continuum. The first is an expanded 5-level scale, a modified version of the Chen scale^19^ (slight wording changes focused on beginning supervision levels with “trusted to…” rather than “allowed to…”), which aligns with undergraduate medical education programs studying the core EPAs for entering residency, which are building blocks for the general pediatrics EPAs. This expanded 5-level scale breaks down full supervision into 2 levels and partial supervision into 3 levels (creating 8 total levels).^19^ The second is a 5-level scale that had been used for a study of fellow performance on 5 EPAs common to both general pediatrics and all subspecialties in pediatrics.^25^ The pediatric GME community, working with the American Board of Pediatrics, created scales for these 5 EPAs to maintain continuity in the transition from residency to fellowship and subsequently demonstrated their reliability in previous work.^25^ As Table 1 illustrates, there are 7 EPAs that are assessed using the 5-level scale and 10 that are assessed using the expanded 5-level scale.

### Data Sources

For each data collection cycle, CCCs at participating sites were asked to submit the following information: levels of supervision for select general pediatrics EPAs, resident postgraduate year, and whether it was spring or fall of the given academic year. Postgraduate year 1 (PGY1) residents at the beginning of the study were included for the duration of the study until they graduated as PGY3 residents at study conclusion. The CCCs were asked to determine levels of supervision using their usual, local CCC decision-making process for assigning milestones. For feasibility purposes, each site was assigned 2 EPAs in common (EPA 5, medical home for well children, and EPA 15, lead an interprofessional team) and randomly assigned to a block with an additional 4 of the 17 general pediatrics EPAs; blocks were balanced to ensure that each EPA other than EPA 5 and EPA 15 was assigned to as near the same number of sites as possible. Sites were not required to report on PGY1 residents in the fall owing to limited experience of and with these trainees. They were also instructed not to report on a resident at any level if they felt unable to assess the EPA.

Emails were sent from the Association of Pediatric Program Directors’ Longitudinal Educational Assessment Research Network to remind site leads to submit data for each cycle. Site investigators were asked to ensure that the same number of CCC members who participated in assigning milestone levels also participated in assigning EPA levels for this study. Residents were assigned study identification numbers prior to data collection, and all data were submitted using only those study identification numbers.

### Bias

All data in this study represent collective entrustment decisions determined by the CCC as a group. The CCC assessment decisions are inherently human-based decisions. Recent evidence suggests this may produce more accurate reflections of performance rather than introduce bias.^38,39^

### Study Size

Sample size calculations required at least 408 learners in total to fit growth curves (described below) and 320 PGY3 learners (across all data collection periods) to achieve 80% power to find that at least 60% of graduating PGY3 learners were rated at the “may perform the EPA without supervision” level on each EPA (vs a null hypothesis that 50% would or would not achieve that level) with *P* < .003 to control for familywise error using the Holm-Sidak procedure.^40^ We determined that distributing EPAs among at least 18 programs with at least 8 residents per training year could provide a sufficient number of PGY3 learners for this analysis for the EPAs assigned to the fewest programs and controlling for clustering of learners within programs. We included additional programs to ensure adequate numbers in the event of attrition.

### Statistical Analysis

We included all data in analyses, and our statistical methods are robust to data missing at random. Because our models include covariates for EPA, PGY, learner, and program, we assume that remaining missingness is at random.

We used descriptive statistics to summarize the points in which 25%, 75%, and 90% of residents achieved each supervision level for each EPA. We fit growth curves to ordinal entrustment levels to make a continuous curve for development in the EPAs that allows one to infer observed development for each month of training. For each EPA, we performed an arcsine–square root transformation of the assigned supervision level relative to the number of levels in the EPA scale (5 for the 5-level scales and 8 for the expanded 5-level scales), treating each scale’s levels as a “proportion of the way toward unsupervised practice”^41^ (which freed us from dependency on the number of scale points). We then modeled the transformed levels for all EPAs together using a linear mixed growth model with fixed effects of resident year (measured in half-years) and year squared (allowing for a nonlinear association between time and supervision), and a random intercept and slope-over-year for the resident, program, and EPA. Statistical significance was set at 2-tailed *P* < .05. Analyses were conducted using R statistical software version 3.4 (R Project for Statistical Computing) and the lme4 package.^42^

We identified the distribution of reported supervision levels reached by the residents at the time of their final CCC review for each of the 17 EPAs. We also identified the percentage of these residents who had reached the level of entrustment for unsupervised practice.

## Results

Across the 5 data collection cycles, 1987 residents from 23 residency programs received 25 503 supervision level reports for the 17 general pediatrics EPAs. The distribution of residents by PGY across data collection cycles is shown in eTable 2 in the Supplement. Across all reporting periods, 14% of single EPA reports (ie, 1 EPA for 1 resident) were determined unable to be assessed. Two programs withdrew during the study period. One of these programs submitted data for the first cycle and the other submitted data for the first 3 cycles. All other programs submitted data for each of the 5 cycles except for 2 programs: 1 did not submit for cycle 1 and 2, and the other did not submit for cycle 1. Data from all programs were included when reported. Comparable numbers of residents in each PGY were assessed in each data collection cycle, with the exception of fall data collections, when PGY1 data were not collected. Most residents were assessed during more than 1 data collection cycle (1 cycle: 799; 2 cycles: 138; 3 cycles: 697; 4 cycles: 78; 5 cycles: 275).

**Figure 1.**
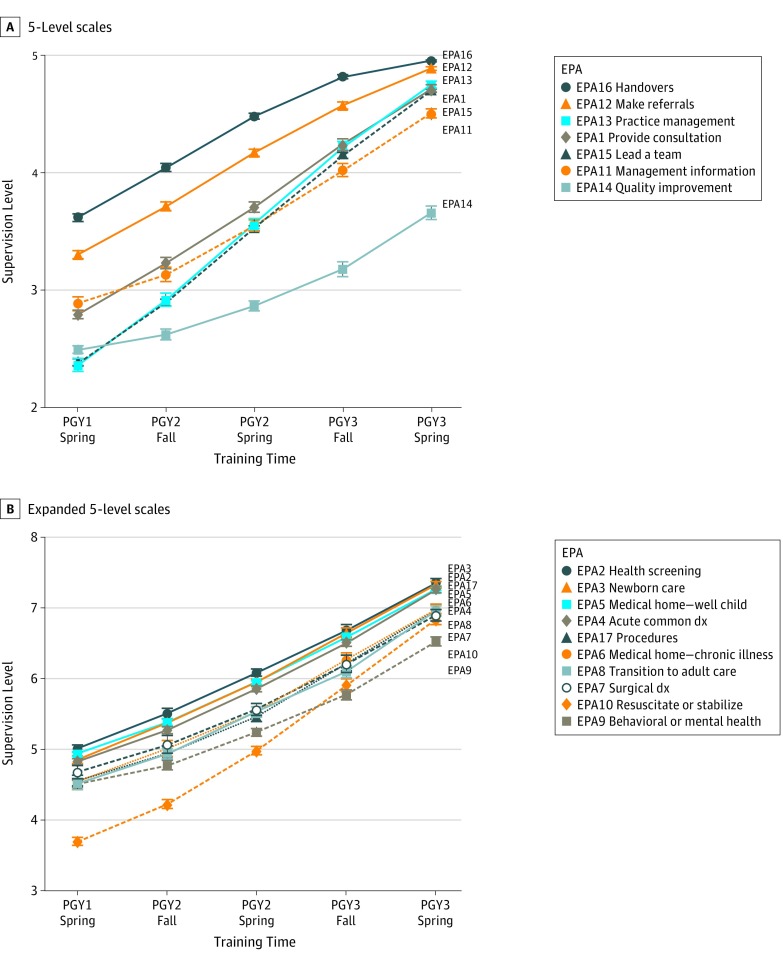
Growth Curves Across Residency for 17 General Pediatrics Entrustable Professional Activities (EPAs) See Table 1 for full description of EPAs and supervision level scales. dx indicates diagnosis; PGY, postgraduate year.

Figure 1 shows the growth curves for all 17 EPAs. The 4 EPAs that required the most supervision across training were EPA 14 (quality improvement) on the 5-level scale (estimated mean level at graduation, 3.7; 95% CI, 3.6-3.7) and EPAs 8 (transition to adult care; mean, 7.0; 95% CI, 7.0-7.1), 9 (behavioral and mental health; mean, 6.6; 95% CI, 6.5-6.6), and 10 (resuscitate and stabilize; mean, 6.9; 95% CI, 6.8-7.0) on the expanded 5-level scale. Compared with mean performance across EPAs on the 5-level scale (4.6; 95% CI, 4.6-4.7), performance for EPAs 12 (refer to consultants; mean, 5.0; 95% CI, 4.9-5.0) and 16 (handovers; mean, 5.0; 95% CI, 5.0-5.0) were significantly higher (*P* < .05 for both). Performance for EPA 14 (quality improvement; mean, 3.7; 95% CI, 3.6-3.7) was significantly lower than mean performance across EPAs (*P* < .05). Finally, performance for EPA 10 (resuscitate and stabilize; mean, 7.1; 95% CI, 6.9-7.3) was significantly lower than mean performance on the expanded 5-level scale.

**Figure 2.**
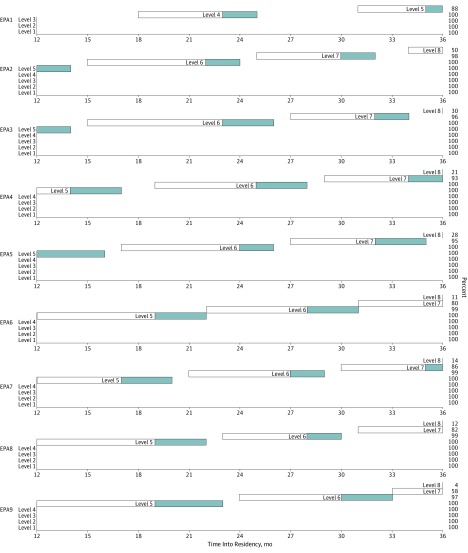
Developmental Progression Scales for General Pediatrics Entrustable Professional Activities (EPAs) 1 to 9 The beginning of the white bar (left side) indicates the point at which 25% of residents achieved a given supervision level, with the end of the white bar (right side) indicating the point at which 75% achieved the level. The end of the blue bar (right side) indicates where 90% of residents achieved the supervision level. See Table 1 for full description of EPAs and supervision level scales.

**Figure 3.**
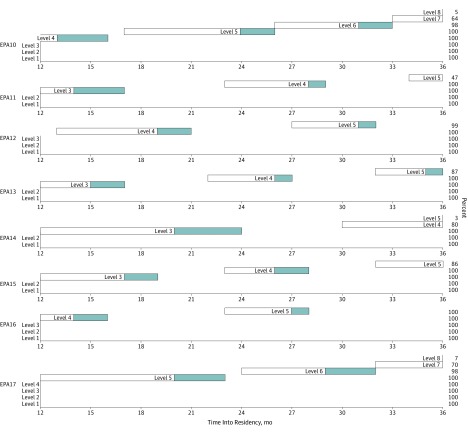
Developmental Progression Scales for General Pediatrics Entrustable Professional Activities (EPAs) 10 to 17 The beginning of the white bar (left side) indicates the point at which 25% of residents achieved a given supervision level, with the end of the white bar (right side) indicating the point at which 75% achieved the level. The end of the blue bar (right side) indicates where 90% of residents achieved the supervision level. See Table 1 for full description of EPAs and supervision level scales.

The EPA Developmental Progression Scales are shown in Figure 2 and Figure 3. The beginning of the white bar (left side) indicates the point at which 25% of residents achieved a given supervision level, with the end of the white bar (right side) indicating the point at which 75% achieved the level. The end of the blue bar (right side) indicates where 90% of residents achieved the supervision level. At 36 months (graduation), trainee development for most EPAs stopped short of 75% (white bar) or 90% (blue bar) of trainees achieving the highest 1 to 2 supervision levels. In these instances, the percentage of residents achieving the highest level is indicated on the right-hand side of the figure. For example, for EPA 9, level 7 ends at 58% of residents and level 8 ends at 4% of residents.

The eFigure in the Supplement shows the percentage of residents achieving each of the supervision levels at the time of graduation (spring report of PGY3-year). For most of the 5-level EPAs, at least 90% of residents achieved at least a level 4 by graduation. These levels correspond with being entrusted to practice with only indirect supervision or no supervision, depending on the EPA (Table 1). For most of the expanded 5-level EPAs, at least 90% of residents received level 6 (supervisor distantly available), 7 (unsupervised), or 8 (trusted to supervise others) by graduation. The lone exception to this is EPA 9 (behavioral and mental health), for which 90% achieved level 5 (“supervisor immediately available and key findings double checked”) or higher.

At the time of graduation (36 months), the percentage of trainees who were rated at a supervision level corresponding to “unsupervised practice” varied by EPA from 53% to 98% (Table 2). If we were to set performance standards that aligned with 90% of trainees achieving the level of unsupervised practice, this standard would be met for only 8 of the 17 EPAs (although 89% met this standard for EPA 17, performing the common procedures of the general pediatrician).

**Table 2.** Percentages of Graduating Residents Reaching Entrustment With Unsupervised Practice

EPA^a^	EPA Abbreviation	No. of Supervision Levels	Level Associated With Entrustment With Unsupervised Practice	Graduating Residents Reaching Entrustment With Unsupervised Practice, % (95% CI)
1	Provide consultation	5	5	69 (62-75)
2	Health screening	Expanded 5	7	93 (89-96)
3	Newborn care	Expanded 5	7	98 (95-99)
4	Acute common diagnosis	Expanded 5	7	93 (89-95)
5	Medical home—well child	Expanded 5	7	91 (89-92)
6	Medical home—chronic illness	Expanded 5	7	79 (74-83)
7	Surgical diagnosis	Expanded 5	7	83 (75-89)
8	Transition to adult care	Expanded 5	7	70 (64-75)
9	Behavioral and mental health	Expanded 5	7	53 (48-59)
10	Resuscitate and stabilize	Expanded 5	7	77 (71-83)
11	Manage information	5	4	92 (88-95)
12	Make referrals	5	5	82 (77-87)
13	Practice management	5	5	63 (55-70)
14	Quality improvement	5	3	90 (85-93)
15	Lead a team	5	4	94 (93-96)
16	Handovers	5	5	94 (90-96)
17	Procedures	Expanded 5	7	89 (84-93)

Abbreviation: EPA, entrustable professional activity.

^a^ See Table 1 for full description of EPAs and supervision level scales.

## Discussion

This study provides initial evidence for using an EPA-based assessment framework to measure the development of clinical skills in residents on a large scale in diverse clinical learning environments over an extended period of time. These findings have implications for GME training programs beyond pediatrics as they suggest that other specialties may be able to implement similar EPA-based assessment systems.

Residents received progressively higher supervision scale ratings for each EPA over time as they gained clinical experience. Furthermore, the rate of growth in supervision scale ratings varied by EPA. This time-dependent (as a marker for gaining experience with the clinical abilities embedded within a given EPA) and EPA-dependent variability in supervision scores provide evidence of construct-relevant variance that supports the validity of using this model as a CBME assessment framework. These findings are similar to those reported in other studies of residents using the ACGME milestones and fellows using the American Board of Pediatrics subspecialty EPAs.^25,43^

For 9 of the 17 EPAs, training programs rated at least 89% of residents as able to practice those EPAs unsupervised by the end of residency training or earlier. However, for the remaining 8 EPAs, a smaller percentage of residents were determined to be able to practice those EPAs unsupervised by the end of their required training. These findings create a dilemma for educators, certifying boards, and other regulatory agencies that will need to be resolved to implement EPA-based assessment more broadly. If we expect residents to meet the standard of unsupervised practice at the completion of training in all 17 EPAs, then either training needs to be enhanced significantly in these areas or our expectations of what residents are required to achieve by the completion of training needs to be adjusted. Future study should determine whether similar experiences, and the related conundrums, are seen in other specialties.

The 4 EPAs with the lowest percentage of residents achieving the level of unsupervised practice by graduation were EPA 1 (provide consultation), EPA 8 (transition to adult care), EPA 9 (behavioral and mental health), and EPA 13 (practice management). For each of these, at least 30% of graduating residents did not meet the level of unsupervised practice. These areas include known gaps in pediatric training and care (eg, transitioning to adult care and behavioral and mental health), suggesting curriculum for training residents in these areas requires notable improvement.^44,45^ For other EPAs, allowing residents to graduate from training programs before they achieve the level of unsupervised practice may be acceptable. For example, for practice management (EPA 13), residency training may not be the best setting to learn this EPA. Perhaps newly graduated residents could engage in structured learning during the early years of continuing certification to meet the standard of unsupervised practice.

Although our data raise concerns and considerations around the development for some EPAs, the vast majority of residents (at least 89%) progressed at a significantly faster rate and met the standards to practice 9 of 17 EPAs unsupervised before reaching the end of training. For at least 1 of these EPAs, EPA 16 (handovers), this may reflect increased opportunities to gain these skills (eg, handovers are extremely common on inpatient services and residents spend significant time coordinating the care of patients with medical complexity) and/or heightened curricular focus due to patient safety and regulatory concerns.^46,47,48^

Our finding that many residents were judged to be ready to practice a range of EPAs without supervision well before completion of residency training and other residents were not ready at the time of graduation makes a strong argument for the shift to time-variable advancement through GME—a system in which outcomes (eg, performance thresholds for EPAs) are fixed and time is a resource to achieve those outcomes. We raise the question: Why not give more responsibility to residents entrusted to perform certain EPAs without supervision? For residents who meet standards early, this could take the form of a period of transition in which they are allowed to practice a given EPA without supervision within the protective environment of residency training and perhaps supervise and teach others in the realm of this professional activity without faculty oversight.^8^ Alternatively, this could shorten the time into general practice or subspecialty training, or allow more flexibility during training to gain additional experience and individualize training for EPAs germane to their career choice, or in other clinical areas where they have not yet met nationally agreed-on graduation standards.

### Limitations

This study has limitations to consider. First, in our analyses, we treat supervision level assignments, which are ordinal, as continuous data. However, this is common and psychometric scholars have noted that “parametric methods can be utilized [in these cases] without concern for ‘getting the wrong answer.’”^49^ Because this study required us to implement the EPA assessment model on a large scale and in complex clinical learning environments, we designed the study to assign each program to implement 2 common EPAs and 4 randomly selected EPAs (6 of 17 EPAs). We did this to enhance feasibility and internal validity of the study. However, this design limits our ability to assess the variability in how programs implemented and assessed the randomly selected EPAs and our ability to generalize these findings across all 23 training programs. It also does not resolve any potential questions regarding programs’ ability to assess residents on all 17 EPAs or assess residents on both EPAs and milestones, which should be the focus of future work. Furthermore, to estimate the rate of growth in supervision scale ratings for each EPA, we extrapolated from data at 5 points during 36 months of residency training rather than from each month of residency training. Therefore, the developmental progression scales we report in Figure 2 and Figure 3 only approximate performance between the reporting periods of this study. However, we believe these scales provide the opportunity to view a dashboard of the rate of progression of a learner against a peer group that may be helpful for providing trainee feedback about strengths and needed areas of focus for improvement. This study sought to understand overall resident performance for the purposes of determining EPA developmental trajectories across a cohort of residents. However, future work should consider individual residents as the unit of analysis.

## Conclusions

This cohort study offers initial evidence of empirically derived competency standards and sets the stage for identifying and filling curricular gaps that account for any discrepancy between the empirically determined standards and those standards needed to produce physicians able to meet the health needs of the patient populations they serve. Future work should include pursuing similar efforts in other specialties.

## Notes

Supplement. **eTable 1.** Association of Pediatric Program Director Longitudinal Educational Assessment Research Network Participating Sites **eTable 2.** Cross-sectional Distribution of Residents Across Data Collection Cycles **eFigure.** Supervision Level at Graduation for 17 General Pediatrics Entrustable Professional Activities
