# Supplementary material for: Longitudinal Assessment of Resident Performance Using Entrustable Professional Activities
Source: JAMA Netw Open. 2020 Jan 15;3(1):e1919316. doi: 10.1001/jamanetworkopen.2019.19316 (PMC6991321; doi:10.1001/jamanetworkopen.2019.19316)
Supplement: Supplement. — eTable 1. Association of Pediatric Program Director Longitudinal Educational Assessment Research Network Participating Sites eTable 2. Cross-sectional Distribution of Residents Across Data Collection Cycles eFigure. Supervision Level at Graduation for 17 General Pediatrics Entrustable Professional Activities [file jamanetwopen-3-e1919316-s001.pdf]

Supplementary Online Content

Schumacher DJ, West DC, Schwartz A, et al; Association of Pediatric Program Directors Longitudinal Educational Assessment Research Network General Pediatrics Entrustable Professional Activities Study Group. Longitudinal assessment of resident performance using entrustable professional activities. *JAMA Netw Open*. 2020;3(1):e1919316. doi:10.1001/jamanetworkopen.2019.19316

eTable 1. Association of Pediatric Program Director Longitudinal Educational Assessment Research Network Participating Sites
eTable 2. Cross-sectional Distribution of Residents Across Data Collection Cycles
eFigure. Supervision Level at Graduation for 17 General Pediatrics Entrustable Professional Activities

This supplementary material has been provided by the authors to give readers additional information about their work.

© 2020 Schumacher DJ et al. *JAMA Network Open.*

eTable 1: Association of Pediatric Program Director Longitudinal Educational Assessment Research Network Participating Sites

Program	Total Categorical Pediatrics Residents Per Study Year Participation
Baylor University/Texas Children's Hospital	2015-2016: 105<br>2016-2017: 106<br>2017-2018: 104
Cincinnati Children's Hospital Medical Center	2015-2016: 120<br>2016-2017: 120<br>2017-2018: 120
Colorado Children's Hospital	2015-2016: 88<br>2016-2017: 88<br>2017-2018: 89
Dartmouth	2015-2016: 21<br>2016-2017: 21<br>2017-2018: Did not participate
Mayo Clinic	2015-2016: 36<br>2016-2017: 36<br>2017-2018: 37
Medical College of Georgia	2015-2016: 45<br>2016-2017: 45<br>2017-2018: 45
Helen DeVos Children's Hospital/ Michigan State University	2015-2016: 42<br>2016-2017: 43<br>2017-2018: 44
New York University	2015-2016: 54<br>2016-2017: 54<br>2017-2018: 54
Rainbow Babies and Children's Hospital	2015-2016: 68<br>2016-2017: 75<br>2017-2018: 74
Rutgers University	2015-2016: 31<br>2016-2017: 28<br>2017-2018: 29
Seattle Children's Hospital	2015-2016: 111<br>2016-2017: 111<br>2017-2018: 118
Stony Brook University	2015-2016: 35<br>2016-2017: 35<br>2017-2018: 35
University of California, Davis	2015-2016: 38<br>2016-2017: 36<br>2017-2018: 36
University of California, San Francisco	2015-2016: 84<br>2016-2017: 80

© 2020 Schumacher DJ et al. *JAMA Network Open.*

	2017-2018: 81
University of Florida	2015-2016: 53<br>2016-2017: 52<br>2017-2018: 50
University of Maryland	2015-2016: 45<br>2016-2017: 45<br>2017-2018: 45
University of Minnesota	2015-2016: 71<br>2016-2017: 73<br>2017-2018: 74
University of North Carolina	2015-2016: 60<br>2016-2017: 58<br>2017-2018: 58
University of Utah	2015-2016: Did not participate<br>2016-2017: 65 (participated in second data collection for year)<br>2017-2018: 66
University of South Dakota	2015-2016: 18<br>2016-2017: 18<br>2017-2018: 18
Virginia Commonwealth University	2015-2016: 47<br>2016-2017: 46<br>2017-2018: 49
Western Michigan University	2015-2016: 23<br>2016-2017: 24<br>2017-2018: 24

© 2020 Schumacher DJ et al. *JAMA Network Open.*

eTable 2: Cross Sectional Distribution of Residents Across Data Collection Cycles

Post-Graduate Year	Spring 2016	Fall 2016	Spring 2017	Fall 2017	Spring 2018
1	353	0	392	0	425
2	346	374	397	385	417
3	276	362	374	363	392

© 2020 Schumacher DJ et al. *JAMA Network Open.*

eFigure. Supervision Level at Graduation for 17 General Pediatrics Entrustable Professional Activities

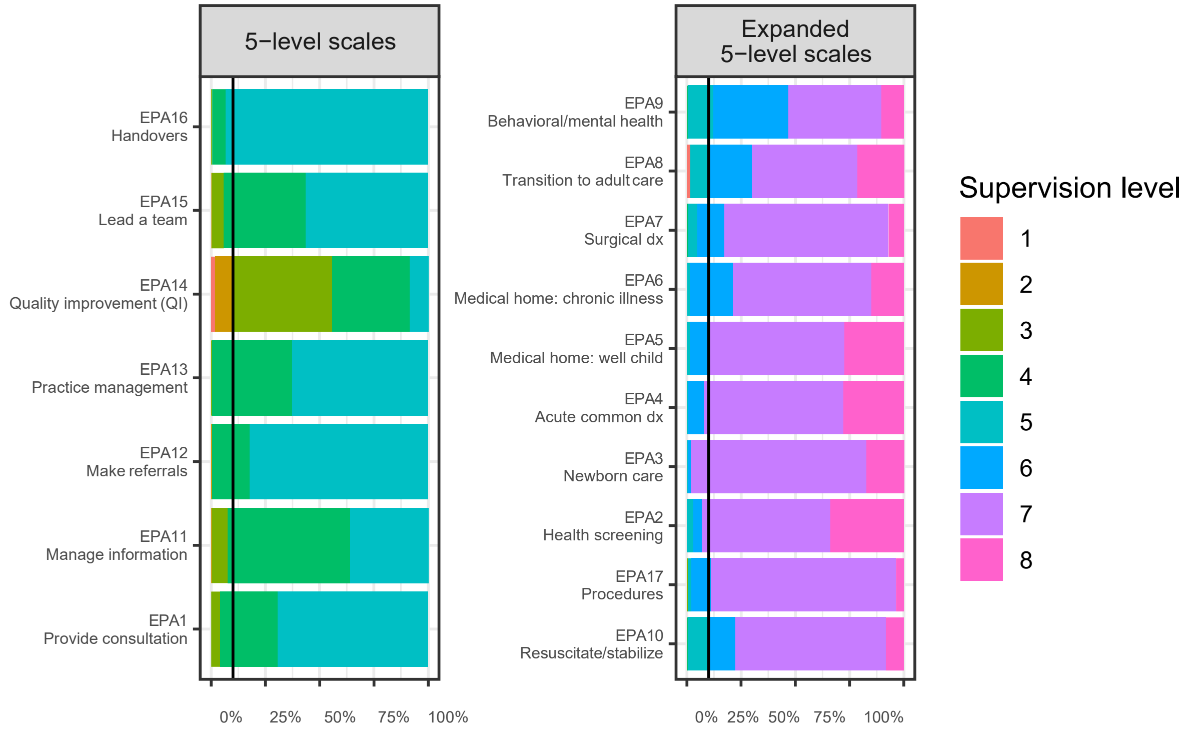

Abbreviations: PGY (post-graduate year), EPA (entrustable professional activity), Dx (diagnosis).

Please see Table 1 in the main article for full description of EPAs and supervision level scales. The black line indicates the level achieved by at least 90% of graduates.

© 2020 Schumacher DJ et al. *JAMA Network Open.*
